# The Plasma Glycoprotein Milieu in the Hemato-Oncological Patient Inhibits Platelet Function

**DOI:** 10.3390/biom16060761

**Published:** 2026-05-22

**Authors:** Iris M. De Cuyper, Graciela Carbajo-Argüelles, María Villa-Fajardo, Andrea Acebes-Huerta, Rutger A. Middelburg, Johannes A. Eble, Dick H. W. Dekkers, Jeroen A. A. Demmers, Jean-Louis H. Kerkhoffs, Jaap Jan Zwaginga, Laura Gutiérrez

**Affiliations:** 1 Sanquin Blood Supply Foundation, Department Research, 1066 CX Amsterdam, The Netherlands; i.decuyper@sanquin.nl; 2 Platelet Research Lab, Instituto de Investigación Sanitaria del Principado de Asturias (ISPA), 33011 Oviedo, Spain; uo157549@uniovi.es (G.C.-A.); andreaacebeshuerta@gmail.com (A.A.-H.); 3 Department of Hematology, Instituto de Investigación Sanitaria San Carlos (IdISSC), Hospital Clínico San Carlos, 28040 Madrid, Spain; maria.vfj5@gmail.com; 4 Center for Clinical Transfusion Research, Sanquin Blood Supply Foundation, 2333 ZA Leiden, The Netherlands; r.a.middelburg@lumc.nl (R.A.M.); j.kerkhoffs@hagaziekenhuis.nl (J.-L.H.K.); j.j.zwaginga@lumc.nl (J.J.Z.); 5 Institute of Physiological Chemistry and Pathobiochemistry, University of Münster, 48149 Münster, Germany; johannes.eble@uni-muenster.de; 6 Center for Proteomics, Erasmus University Medical Center (Erasmus MC), 3015 CN Rotterdam, The Netherlands; d.dekkers@erasmusmc.nl (D.H.W.D.); j.a.a.demmers@lacdr.leidenuniv.nl (J.A.A.D.); 7 Department of Medicine, University of Oviedo, 33006 Oviedo, Spain; 8 Centro de Investigación Biomédica en Red de Salud Mental (CIBERSAM), 28029 Madrid, Spain

**Keywords:** platelet function, platelet transfusion, hemato-oncological patients, chemotherapy, plasma factors, vWF

## Abstract

Hemato-oncological patients with chemotherapy-induced thrombocytopenia are a major recipient group of frequent platelet (PLT) transfusion. Prophylactic platelet transfusions are administered when platelet counts fall below 10 × 10^9^ PLT/L, to prevent severe or fatal bleeding. However, these prophylactic platelet transfusions do not always result in the prevention of bleeding. Pre- or post-transfusion acquired dysfunction of donor platelets in this respect could play a role. We previously reported intrinsic and transfusion-dependent platelet alterations in hemato-oncological patients. In particular, the expression of relevant platelet receptors was affected in donor platelets after incubation with patient’s plasma, which could explain, at least in part, the variable efficacy of platelet transfusions in these patients. In the present manuscript we show that plasma from acute myeloid leukemia (AML) patients undergoing chemotherapy inhibits functionality of allogenic platelets. Further proteomic analysis allowed us to observe alterations in the composition of plasma samples, and to identify key plasma components which could be responsible for platelet function inhibition and explain bleeding in patients notwithstanding platelet transfusions. We anticipate that with the obtained results, platelet transfusion support can be further personalized in patients receiving chemotherapy and applications might expand to maximize the clinical efficacy of procedures such as bone marrow transplantation.

## 1. Introduction

Hemato-oncological patients with chemotherapy-induced thrombocytopenia are one of the major populations receiving frequent platelet (PLT) transfusions [1,2]. The management and general guidelines associated with these patients, although subject to local hospital guidelines, include administering prophylactic platelet transfusions when platelet counts fall below 10 × 10^9^ PLT/L, rather than waiting for the need of therapeutic transfusions (i.e., due to bleeding), because they are regarded to have an increased risk of clinically relevant bleeding [3]. Still, bleeding during prophylactic transfusion regimens occurs frequently, also lacking a clear correlation with platelet count as well as platelet corrected count increment (CCI) [4,5]. The reasons for this variability in bleeding and platelet efficacy in hemato-oncologic patients are still largely elusive [6,7].

Chemotherapy in this respect not only inhibits hematopoiesis causing anemia and thrombocytopenia, but it also has adverse effects on coagulation, inflammatory cells, and the vascular wall [8,9,10]. All these factors may cause platelet activation, which could favor increased platelet clearance and a remanent anergic platelet population in circulation. Disturbed platelet function has been reported in cancer patients receiving chemotherapy, regardless of the platelet count, leading to either hemorrhagic or thrombotic consequences [11]. Vinholt et al. showed that the aggregation capacity of platelets from acute myeloid leukemia (AML) and myelodysplastic syndrome (MDS) patients with a bleeding tendency, in the presence of healthy donor plasma, after stimulation with ADP, TRAP-6 and collagen-related peptide (CRP) was reduced [12]. The lower aggregation capacity of platelets in AML associated with bleeding in their studies suggested that the functional assessment of platelets could be a bleeding predictor, beyond the platelet count. In a later study, the authors confirmed their previous findings, and observed that various regimens of platelet transfusion would not alter the observed platelet functional unbalance [13]. Moreover, thrombocytopenia is not sufficient to cause bleeding as there are pathophysiological alterations that may contribute to it; for example, the presence of activated leukocytes mediating vascular damage is required [14].

In previous studies, we observed two categories of immunophenotypic platelet alterations in hemato-oncological patients, which we tentatively named “underlying”, referring to the alterations present in platelets from the patient, and “transfusion-dependent”, referring to the alterations inflicted on donor platelets after transfusion. Interestingly, incubation of platelets from healthy donors with plasma from AML patients affected the expression of relevant platelet receptors, either through shedding or recycling, which could subsequently cause platelet dysfunction, explaining, at least in part, the variable efficacy of platelet transfusions in these patients [15]. While this study could not discern a potential contribution of the donor platelet basal status (i.e., platelet storage lesion) to the “transfusion-dependent” alterations observed, results clearly support that the plasma of hemato-oncological patients interferes with platelet quality or integrity. Supporting this notion, a recent characterization of the composition of plasma from AML patients revealed a disbalance in proteins involved in inflammation and complement system, endopeptidase inhibitors activity, lipoprotein remodeling, and coagulation, which may contribute to the bleeding risk acknowledged in AML patients [16].

We hypothesize that the pathophysiological environment of the plasma milieu actively influences the efficacy of platelet transfusions in chemotherapy-treated hemato-oncological patients. In the present study we aimed at studying whether plasma from AML patients could alter the functionality of platelets from healthy donors, using a flow cytometry-based platelet aggregation test developed by us (FCA) [17], with the objective to identify the responsible plasma alterations. Many key regulators of hemostasis are plasma glycoproteins, including von Willebrand factor (vWF), fibrinogen, fibronectin, complement components, and immunoglobulins [18,19]. Glycosylation critically modulates plasma protein stability, receptor recognition, multimer formation, and integrin binding capacity [20,21]. Furthermore, hematologic malignancies and systemic inflammatory states are associated with quantitative and qualitative alterations in plasma glycosylation patterns, which can modify the biological function of circulating proteins [21,22]. In AML, dysregulation of acute-phase proteins, complement pathways, and hemostatic factors have been reported [23]. Therefore, assessment of global plasma glycoprotein patterns was designed as an exploratory approach to identify alterations in the AML plasma milieu that could mechanistically contribute to platelet functional inhibition.

Delineation of how such novel determinants of hemostasis apply to chemotherapy patients is essential to first understand the variable effectivity of platelet transfusions in the mentioned patient population and eventually to progress towards more individualized strategies for platelet support, i.e., based on risk factors apart from merely the platelet count.

## 2. Materials and Methods

### 2.1. Study Population

Plasma samples collected from patients with acute myeloid leukemia (AML), included in the Pathogen Reduction Evaluation and Predictive Analytical Rating Score (PREPAReS) trial, were used [24]. Clinical data of patients whose samples were included in the study are indicated in Table S2.

The study was approved by our institute’s (Sanquin) medical ethics committee in accordance with the 1964 Declaration of Helsinki.

### 2.2. Sample Collection and Processing

Citrate-anticoagulated blood samples from AML patients were centrifuged at 210× *g* for 15 min (no brake, room temperature), and the upper three quarters of the platelet rich plasma fraction were collected into a new tube, and centrifuged at 2310× *g* for 5 min (no brake, room temperature). The platelet-poor plasma fraction was transferred into a new tube, centrifuged at 10 krpm to remove cell debris and transferred into clean tubes. Plasma samples were snap frozen and kept at −80 °C until further use.

A pool of 5 blood group AB Rhesus D-positive (AB RhD+) plasmas from healthy donors (CPD plasma) was used as control (obtained from Sanquin Blood Bank).

Heparin-anticoagulated blood samples from blood group 0 Rhesus D-positive (0 RhD+) healthy donors were used to obtain washed platelets for platelet function testing, after informed consent was obtained. In brief, blood samples were centrifuged at 210× *g* for 15 min (no brake, room temperature), and the upper three quarters of the platelet-rich plasma fraction were collected into a new tube, and centrifuged at 2310× *g* for 5 min (no brake, room temperature). Platelets were washed twice with sequestrine buffer (17.5 mM Na_2_HPO_4_, 8.9 mM Na_2_EDTA, 154 mM NaCl, pH 6.9, containing 0.1% [wt/vol] bovine serum albumin) by centrifuging them at 2310× *g* for 5 min. Platelets were resuspended to a final concentration of 50 × 10^6^ PLT/mL in HEPES-Glucose buffer solution (132 mM NaCl, 6 mM KCl, 1 mM MgSO_4_, 1.2 mM KH_2_PO_4_, 20 mM HEPES, pH 7.4, containing 5 mM glucose).

### 2.3. Flow Cytometry-Based Platelet Aggregation Assay (FCA)

The flow cytometry-based platelet aggregation assay (FCA) was performed as previously described [17]. In brief, reactions were set with 5 × 10^4^ PLT/µL, in a final volume of 100 µL HEPES-Glucose buffer solution containing 20% of patient or AB RhD+ donor plasma, as indicated. Platelets were isolated from blood group 0 RhD+ donors to minimize incompatibility. Platelets were divided into two fractions and incubated with either CD31-APC or CD31-PE (BD Biosciences; San Jose, CA, USA) antibodies. The two fractions were then mixed at 1:1 to set the aggregation reactions. Next, 3 mM CaCl_2_ were added prior to inducing platelet aggregation. Agonists used were ristocetin (1.5 mg/mL), phorbol 12-myristate 13-acetate (PMA,100 ng/mL), type-I collagen (10 µg/mL), convulxin (CVX, 2 µg/mL) and aggretin A (AggA, 30 µM). Reactions were performed on round-bottom Eppendorf tubes (2 mL), at 1000 rpm, 37 °C on a Thermomixer (Eppendorf; Hamburg, Germany). To construct an aggregation curve, 10 µL were taken at 30 s, 1 min, 2 min, 5 min and 10 min, and resuspended in 140 µL of 0.5% (vol/vol) formaldehyde in PBS prior to measuring by flow cytometry. Data was analyzed with FlowJo v10, as described [17]. From each aggregation reaction (see representative reactions in Figure S1) we obtained the area under the curve (AUC—the extent of the response) and the time of maximum aggregation (TMA) for further analysis.

### 2.4. vWF and Fibrinogen ELISA

vWF ELISA was performed as previously described, using a SkanWasher 40 for the washing steps and a SpectraMax Plus 384 for measuring at 450 nm with 540 nm as a reference wavelength (both from Molecular Devices; San José, CA, USA) [25,26,27,28]. In particular, at least four dilutions of plasma (range 1:20–1:5000) were measured in duplicate from each sample, and after discarding outliers above the standard curve, the average was taken as sample concentration. Control samples used were obtained from healthy donor independent plasma units from Sanquin Blood Bank. Fibrinogen ELISA (Cloud-Clone Corp.; Houston, TX, USA; #SEA193Hu) was performed following the manufacturer’s instructions.

### 2.5. SDS-PAGE Electrophoresis and Glycoprotein Staining

Plasma samples were prepared with Laemmli loading buffer and preheated at 65 °C 5 min before being run on SDS-PAGE gels (4–15% Mini-PROTEAN^®^ TGX™ Precast Protein Gels, Bio-Rad; Hercules, CA, USA) and glycoproteins were stained with PierceTM Glycoprotein Staining kit (Thermo Scientific; Waltham, MA, USA). Subsequently, the indicated bands (Figure S1) were cut and processed for mass spectrometry analysis.

### 2.6. Mass Spectrometry Analysis

In-gel digestion of the selected glycoprotein-stained gel bands was carried out using a protocol similar to that used for Coomassie-stained gels, as described [29]. No noticeable adverse effects of the glycoprotein staining were observed.

SDS-PAGE gel lanes were cut into 2 mm slices and subjected to in-gel reduction with dithiothreitol, alkylation with iodoacetamide and digested with trypsin (sequencing grade; Promega), as described previously [29]. Nanoflow liquid chromatography tandem mass spectrometry (nLC-MS/MS) was performed on an EASY-nLC coupled to an Orbitrap Fusion Tribid mass spectrometer (Thermo Scientific) or an Orbitrap Fusion Tribid mass spectrometer (Thermo Scientific), both operating in positive mode. Peptides were separated on a ReproSil-C18 reversed-phase column (Dr. Maisch HPLC GmbH; Ammerbuch, Germany; 15 cm × 50 μm) using a linear gradient of 0–80% acetonitrile (in 0.1% formic acid) during 90 min at a rate of 200 nl/min. The elution was directly sprayed into the electrospray ionization (ESI) source of the mass spectrometer. Spectra were acquired in continuum mode; fragmentation of the peptides was performed in data-dependent mode by HCD. Peak lists were automatically created from raw data files using the Mascot Distiller software (version 2.3; MatrixScience, Boston, MA, USA). The Mascot search algorithm (version 2.2, MatrixScience) was used for searching against the Uniprot proteome reference database (up_human_9606_2017_10.fasta). The peptide tolerance was typically set to 10 ppm and the fragment ion tolerance was set to 0.8 Da. A maximum number of 2 missed cleavages by trypsin were allowed and carbamidomethylated cysteine and oxidized methionine were set as fixed and variable modifications, respectively. The Mascot score cut-off value for a positive protein hit was set to 60. Individual peptide MS/MS spectra with Mascot scores below 40 were checked manually and either interpreted as valid identifications or discarded. Typical contaminants, also present in immunopurifications using beads coated with pre-immune serum or antibodies directed against irrelevant proteins were omitted from the table. Data is shown in Table S1.

### 2.7. Statistics

Platelet aggregation data was analyzed using R platform/environment (version 4.5.2).

Platelet aggregation data processing: The analysis was done in batches, with each batch having its own day-control plasma. Data was normalized by setting the average of AUCs obtained per agonist on control samples to 100. TMA values were processed without normalization.

Results are expressed as mean ± standard deviation (SD). Comparisons between conditions (Control vs. Patient) were performed using Student’s *t*-test, with statistical significance set at *p* < 0.05 (*), *p* < 0.01 (**) and *p* < 0.001 (***). Linear regression was used to analyze the effect of fibrinogen and vWF levels on the TMA of reactions induced by PMA or Ristocetin. Statistical analysis and figures were generated using R environment version 4.5.2 (R Core Team, 2026) [30] and Microsoft Excel. Figures were designed using Adobe Illustrator CS5 (version 15.0). All data are shown in Table S2.

## 3. Results

### 3.1. Plasmas from Hemato-Oncological Patients Alter the Aggregation Capacity of Healthy Donor Platelets Towards Different Agonists

In order to elucidate whether the plasma from hemato-oncological patients has modulatory effects on healthy donor platelet function, we performed platelet aggregation assays using compatible healthy donor platelets in the presence of plasma samples from AML patients and measuring the platelet aggregation capacity to a battery of agonists: collagen (activates GPVI and integrin α2β1), the snake venom convulxin (activates GPVI), the snake venom Aggretin A (activates CLEC2), PMA (activates integrin αIIbβ3) and Ristocetin (which induces platelet agglutination via the vWF receptor, -vWF R-). As control, all reactions were set in parallel with healthy donor platelets in the presence of AB RhD+ plasma obtained from healthy donors.

**Figure 1:**
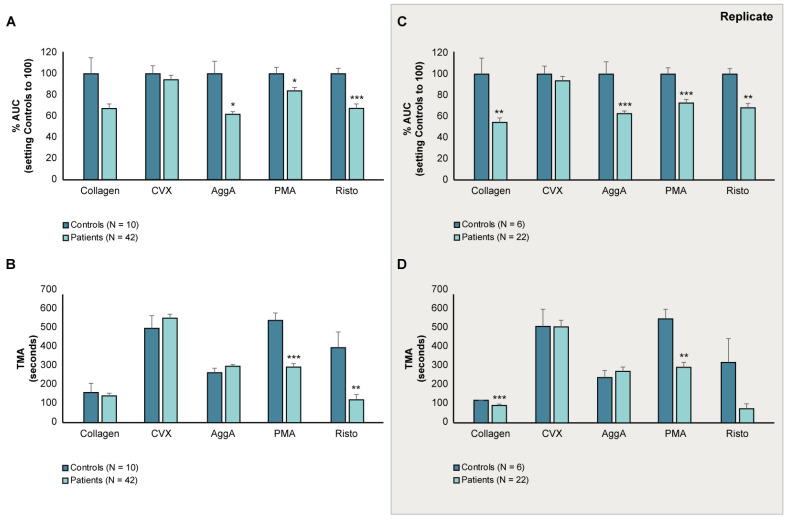
Inhibition of healthy donor platelet aggregation in the presence of AML patient plasma. (**A**,**C**) Bar graphs representing the healthy donor platelet aggregation response (area under the curve, AUC) per agonist in the presence of AML patient plasma or AB RhD+ control plasma. The AUC average in controls was set to 100, per agonist, for better visualization. (**B**,**D**) Bar graphs representing the healthy donor platelet aggregation response (time of maximum aggregation, TMA) per agonist in the presence of AML patient plasma or AB RhD+ control plasma. Graphs within the grey square (**C**,**D**) depict results from a replicate of the experiment shown in (**A**,**B**) using a subset of the original samples. The average and standard deviation is represented. CVX, convulxin; AggA, aggretin A; PMA, Phorbol 12-myristate 13-acetate; Risto, ristocetin. * *p* < 0.05, ** *p* < 0.01, *** *p* < 0.001.

When plotting the aggregation responses per agonist, as shown in the bar graph in Figure 1A, we observed that the plasmas of AML patients partially inhibited the response of healthy donor platelets towards all agonists except convulxin (GPVI-mediated responses) and collagen, the latter showing only a trend toward reduction in the presence of AML plasma (N = 42) compared to control plasma (N = 10). The tendency of inhibition of healthy donor platelet responses towards collagen, while the response towards convulxin was unaffected, suggests, in average, a specific modulation of the integrin α2β1-mediated response, and not GPVI. Interestingly, when plotting the obtained TMAs, we observed that the donor platelets responded significantly faster in response to PMA and Ristocetin (that target αIIbβ3 and vWF-R, respectively) in the presence of AML plasma (Figure 1B, also see Figure S1 and Table S2).

These results were confirmed by replicating the experiments with a selection of AML and control plasmas on independent platelet healthy donor samples (Figure 1C,D). In the replicate, the reduction in aggregation in response to all agonists used, except convulxin, was significant in the presence of AML plasma (N = 22) compared to control plasma (N = 6) (Figure 1C). The TMAs observed followed the same pattern, although the faster response to Ristocetin was not significant in these replicas, as opposed to the TMA upon collagen stimulation, which was subtly but significantly faster (Figure 1D and Table S2).

Overall, our results suggest that components present (or absent) in the plasma of AML patients are potentially responsible for the observed unbalance of platelet aggregation profiles, as exemplified by the reduced aggregation capacity towards almost all agonists tested and the accelerated response towards the stimulation of the so-called hemostatic receptors αIIbβ3 and vWF-R. These results suggest, in potency, that the patient environment where healthy donor platelets will circulate after transfusion of platelet concentrates may modulate platelet responses and reactivity capacity, contributing to the variable efficiency of platelet transfusions in AML patients regarding bleeding.

### 3.2. The Inhibitory Effect of Plasmas from Hemato-Oncological Patients Is Not Overcome upon Transfusion

**Figure 2:**
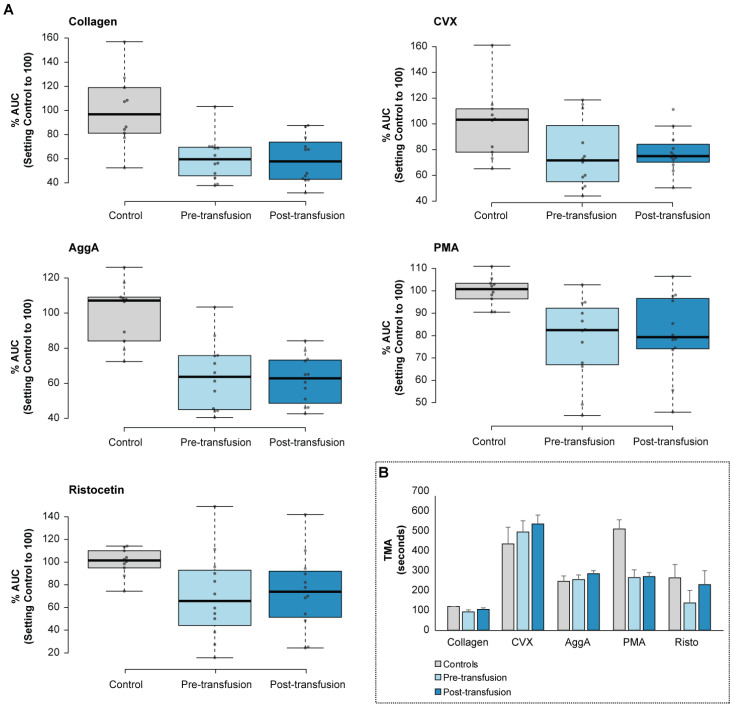
Inhibition of healthy donor platelet aggregation in the presence of AML patient plasma pre- and post-transfusion. (**A**) Box plots representing the healthy donor platelet aggregation response (area under the curve, AUC) per agonist in the presence of AML patient plasma, pre- and post-transfusion (N = 12 pairs), or AB RhD+ control plasma (N = 10). The AUC average in controls was set to 100, per agonist, for better visualization. Center lines show the medians; box limits indicate the 25th and 75th percentiles as determined by R software; whiskers extend 1.5 times the interquartile range from the 25th and 75th percentiles. (**B**) Bar graph representing the healthy donor platelet aggregation response (time of maximum aggregation, TMA) per agonist in the presence of AML patient plasma, pre- and post-transfusion, or AB RhD+ control plasma. CVX, convulxin; AggA, aggretin A; PMA, Phorbol 12-myristate 13-acetate; Risto, ristocetin.

Since platelet transfusions are frequent in these patients, we next aimed at studying directly whether the inhibitory effects of patient plasmas would be modulated upon platelet transfusion (e.g., by adsorption or additional release of modulators). For this purpose, we collected plasma samples before and <4 h after transfusion, and compared paired-wise the platelet aggregation responses, as described above. We could not identify significant changes in the plasma-induced inhibitory responses towards the battery of agonists employed regarding the AUC (Figure 2A). Interestingly, and although it did not reach statistical significance, we observed that, in the presence of AML plasma post-transfusion and compared to pre-transfusion AML plasma samples, there was a tendency of restoration of the TMA upon stimulation with Ristocetin (Figure 2B).

Globally, there were not statistically significant changes when integrating the results from the pre- and post-transfusion paired samples; however, Ristocetin-induced responses were the ones with a higher chance of being partially improved post-transfusion, at least considering the response kinetics.

### 3.3. Healthy Donor AB RhD+ Plasma Partially Rescues the Ristocetin Response Defects Induced by Plasmas from Hemato-Oncological Patients In Vitro

**Figure 3:**
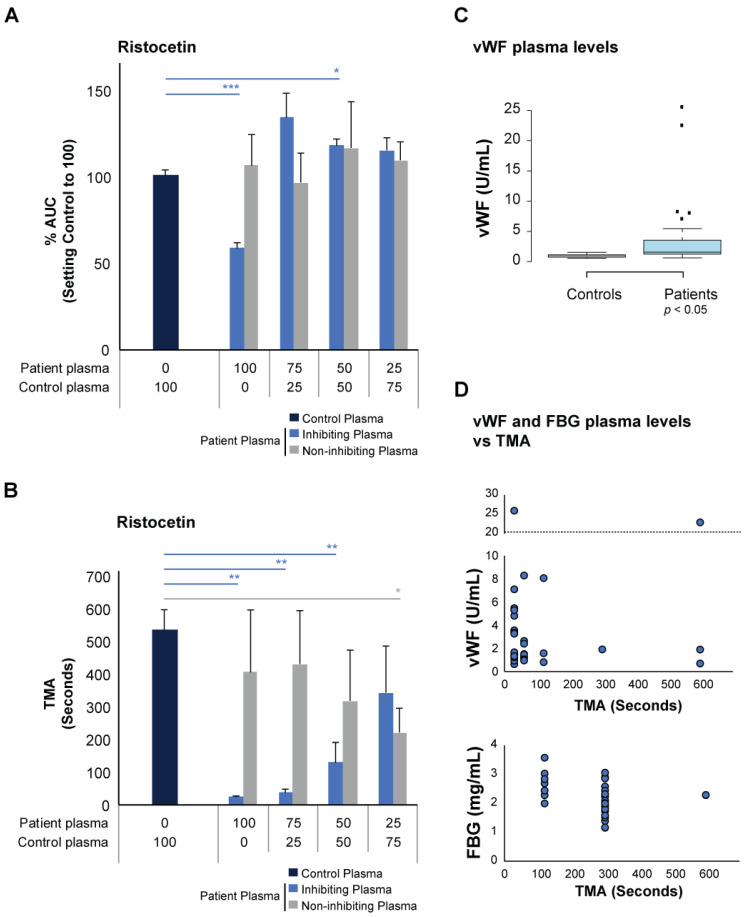
vWF and Ristocetin-mediated platelet aggregation responses. Bar graphs representing the healthy donor platelet aggregation response. The area under the curve, AUC, (**A**) and the time of maximum aggregation, TMA, (**B**) in the presence of plasmas from AML patients that inhibit (blue bars, N = 4) or do not inhibit (gray bars, N = 3) the Ristocetin-mediated platelet aggregation response, with serial dilutions with AB RhD+ control plasma (100/0; 75/25; 50/50; 25/75; 0/100) are depicted. The dark blue bar on each graph shows data from aggregation reactions performed with control plasma only (N = 5). (**C**) Box plot depicting vWF concentration as measured by ELISA in AML patient plasma samples and AB RhD+ control plasmas. Center lines show the medians; box limits indicate the 25^th^ and 75^th^ percentiles as determined by R software; whiskers extend 1.5 times the interquartile range from the 25th and 75th percentiles. (**D**) Dot plots depicting the plasma concentration of vWF against the respective TMA obtained in the aggregation reactions towards Ristocetin stimulation (top, N = 33); the plasma concentration of fibrinogen (FBG) against the respective TMA obtained in the aggregation reactions towards PMA stimulation is shown in the bottom (N = 31). Dotted line marks the gap in the Y axis. * *p* < 0.05, ** *p* < 0.01, *** *p* < 0.001.

We next set out to examine in vitro whether addition of healthy donor plasma (AB RhD+) could specifically modulate or counteract the effects of those patients’ plasmas inhibiting the Ristocetin-mediated response. For that purpose we designed an experiment in which we selected four plasmas with a Ristocetin inhibitory effect, and three plasmas without a Ristocetin inhibitory effect, and prepared aggregation assays with serial dilutions of the patient’s plasma with AB RhD+ healthy donor plasma (100:0, 75:25, 50:50, 25:75, 0:100). As shown in Figure 3A, a proportion of 25% donor plasma vs. 75% patient plasma was already sufficient to counteract the inhibitory effects of patient plasmas on the Ristocetin-mediated response, which was maintained unaltered with increasing proportion of control AB RhD+ plasma. Serial dilutions of patient plasma with healthy donor AB RhD+ plasma, when the plasma had no inhibitory effect on the Ristocetin-mediated response, had no effect whatsoever on the obtained AUCs (Figure 3A). Regarding the TMA, we also observed a restoration, which was reached with a proportion of 75% donor plasma. Of note, the TMA was also affected paradoxically in increasing dilutions of plasmas with no inhibitory effect with healthy donor AB RhD+ plasma (Figure 3B).

Overall, these results suggest that, potentially, the healthy donor plasma might be both providing something that is deficient or lacking in the patient plasma, or diluting/remodeling stimulatory or inhibitory components (such as increased vWF), which both can restore Ristocetin-mediated platelet agglutination to control values (Figure 3A,B).

These data led us to think that this phenomenon could be associated with alterations in vWF plasma levels in AML patients, since vWF is also an acute-phase biomarker of systemic inflammation. Therefore, we assessed vWF plasma levels by ELISA in AML patients. In fact, vWF levels were increased (with different grades of extent) in AML patient plasma samples (Figure 3C), with, in this respect, possible potentiation of Ristocetin-mediated responses. Next, vWF and fibrinogen plasma levels were plotted against the TMAs obtained in the aggregation reactions upon Ristocetin and PMA stimulation, respectively. The dot plots show that there was a tendency for faster responses associated with higher plasma levels of each factor (Figure 3D). Linear regression analyses were performed on these data, and while we found no significance when comparing vWF levels with the TMA of Ristocetin-induced aggregation responses, nor comparing fibrinogen levels with the PMA-induced aggregation responses, significance was observed with vWF levels and the TMA of PMA-induced aggregation (*p* < 0.05). Consistently, multiple linear regression analysis revealed that vWF and fibrinogen plasma levels were significantly associated with the TMA of PMA-induced responses (*p* < 0.05).

### 3.4. Distinct Plasma Glycoprotein Pattern in AML Patients Associated with the Inhibitory Effects on the Aggregation of Healthy Donor Platelets

**Figure 4:**
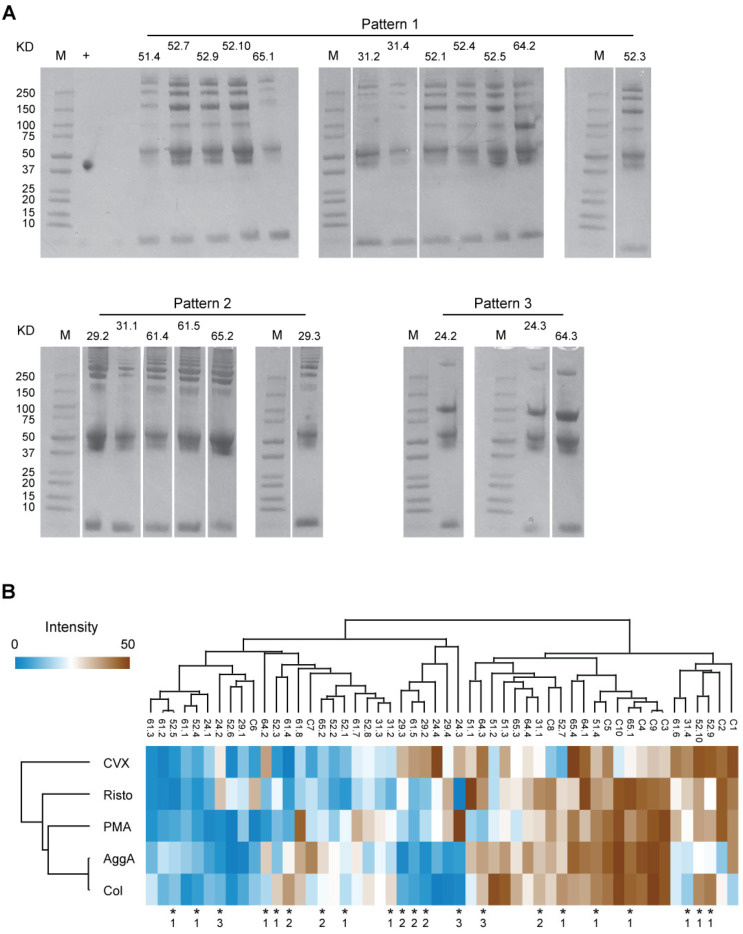
Plasma glycoprotein profiling and cluster analysis of platelet responses. (**A**) Gel photographs of selected plasma samples run on SDS-PAGE gels and stained for glycoproteins. Three different patterns were observed: Pattern 1, similar to the pattern observed in control plasmas [31], and Patterns 2 and 3. (**B**) Cluster visualization of the healthy donor platelet aggregation responses in the presence of AML patient plasma or AB RhD+ control plasma. With an asterisk, the plasmas with characterized glycoprotein pattern are indicated. M, marker; KD kilodalton; Col, collagen; CVX, convulxin; AggA, aggretin A; PMA, Phorbol 12-myristate 13-acetate; Risto, ristocetin.

We next set out to examine whether the plasma glycoprotein pattern would differ among AML patients with different inhibitory effects. This analysis allowed us to identify three distinct glycoprotein patterns. Pattern 1, which is similar to the pattern of plasma from healthy donors [31], and Patterns 2 and 3, that displayed altered patterns, with either band-intensity alterations, or with bands missing or appearing (Figure 4A). Pattern 2 is characterized by loss of the 100 KD band, and appearance of high-molecular-weight bands above 250 KD. Pattern 3 is characterized by intensification of the 100 KD band, and disappearance of the 250 KD and 150 KD bands.

We next performed a hierarchical clustering of the platelet aggregation profiles obtained with patient plasmas and control plasmas. The selected plasmas in which the glycoprotein pattern had been studied are indicated. While there was not a clear separation of the aggregation profiles obtained in the presence of plasma from AML patients based on their glycoprotein pattern, we observed two main clusters. As shown in Figure 4B, the cluster on the right contains the aggregation profiles obtained with all control plasmas but one, with half of the AML plasmas with Pattern 1, and with one AML plasma with Pattern 3. The cluster on the left contains the aggregation profiles obtained with AML plasmas belonging to Pattern 2, which were the ones with a more distinct profile compared to controls (Figure 4B). The remaining aggregation profiles obtained with AML plasmas with Patterns 1 and 3, and one control, also clustered together in the latter. These results suggest that the glycoprotein pattern is potentially associated with the inhibitory effect of the plasma from AML patients on platelets from healthy donors.

### 3.5. Mass Spectrometry Analysis Reveals Differences in Patient Plasma Glycoprotein Milieu

We next decided to analyze by mass spectrometry the proteins contained in differentially appearing bands from Patterns 2 and 3 (clearly associating with plasma inhibitory effects on donor platelet functionality), compared to Pattern 1, which is similar to control plasma. In particular, we cut gel slices from the 10 bands observed in a plasma sample with Pattern 1 (52.9, samples 1–10), and samples a–g correspond to bands present in plasma samples with Patterns 2 and 3 (Figure 2 and Figure S2).

A total of 766 proteins were identified across samples. For data analysis, we discarded identified proteins with a Mascot score < 100 across samples which filtered down the identified proteins across samples to 425. We next compared the identified proteins among Pattern 1 and Patterns 2 and 3 of AML plasma samples corresponding to gel bands of similar molecular weight, as follows: bands 1–2 (Pattern 1) with bands a–d (Pattern 2); bands 3–5 (Pattern 1) with bands, e.g., (Patterns 2 and 3). Of note, we considered protein detection for protein hits above 100 Mascot Score. These comparisons allowed us to select common entries across Patterns (161 proteins), and unique entries to either Pattern 1 (200 proteins) or Patterns 2 and 3 (64 proteins), (see Figure S2 and Table S1).

**Figure 5:**
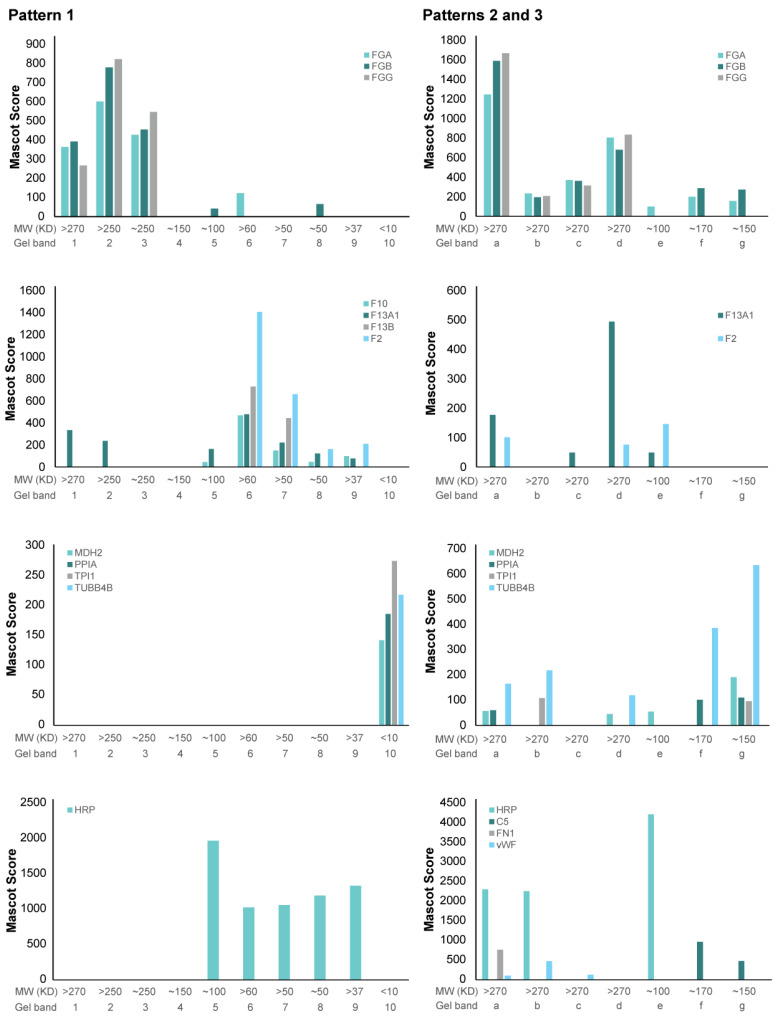
Targeted mass spectrometry analysis. Bar graphs representing selected protein hits as identified by targeted mass spectrometry analysis. All bands present in Pattern 1 (1 to 10, (**left**)) and selected gel bands from Patterns 2 and 3 (**right**) were processed and analyzed (Gel band numbers or letters are as indicated in Figure S1). Obtained Mascot Scores are represented, and the gel band and approximate corresponding molecular weight (MW) is indicated. At the top, Fibrinogen A–C, and identified coagulation factors are depicted. The third and fourth row depict proteins running at higher MW bands in Patterns 2 and 3, compared to control. The fourth row also depicts proteins solely identified in Patterns 2 and 3 (C5, FN1 and vWF).

First, we examined the detection profile of fibrinogen and coagulation factors detected in Pattern 1 plasma. As seen in Figure 5, the detection profile was as expected, with fibrinogen (FBA, FBB, FBG) being detected in high-molecular-weight bands, and coagulation factors (F2, F10, F13A1 and F13B) being detected in mid-molecular-weight bands. In Patterns 2 and 3 (selected bands, of high-molecular-weight as indicated), the detection of fibrinogen appeared normal; however, we could detect F2 in bands of higher molecular weight, which was not the case in Pattern 1 plasma. With similar behavior as F2, we could identify other proteins in the bands examined in Patterns 2 and 3 located in lower-molecular-weight bands in Pattern 1, as if they were displaced within a complex or modified, such as HRP, MDH2, PPIA, TPI1 and TUBB4B (Figure 5 and Table S1).

Interestingly, proteins that were detected solely in the differentially appearing bands, in Patterns 2 and 3, but that were absent in the proximal bands from Pattern 1 included vWF, FN1, cytoskeletal proteins or proteins involved in vesicle transport, and proteins that belong to the complement system (see Figure 5 and Table S1).

On the other hand, proteins that were absent in Patterns 2 and 3, compared to proximity bands from Pattern 1 included proteins regulating cholesterol transport or those involved in cell adhesion. Particularly, focusing first on the 100KD band not visible in Pattern 2, one of the proteins contained in this band in Pattern 1 is APOA4, which has been described to inhibit platelet function through direct interaction with the αIIbβ3 integrin [32]. The triad of ALCAM, MCAM, NCAM2 has been described as emphasizing the role of platelets as neuronal and innate immune cells [33]. CDH13, an adiponectin receptor, is another of these proteins; while no direct association of this protein with platelet functionality has been reported, it is associated with cardiovascular risk, and adiponectin modulates platelet function [34,35]. DPP4 activity loss has been linked to an enhanced adherence of platelets to endothelial cells under flow conditions [36]. LYVE1 is connected to the role of platelets in the lymphatic vessel development and maintenance [37]. PLTP interacts with platelets, promoting their aggregation and influencing blood clotting by altering membrane phospholipids, creating surfaces for coagulation factors, and potentially affecting thrombosis [38].

Bands in Pattern 1, but missing in Pattern 3 (150 and 250 KD), contain ADIPOQ, which has been shown to inhibit platelet activation [39]: APOA4 (described above); ENO1, whose role in coagulation is significant, particularly in patients with cancer, where it may influence tumorigenesis and progression by affecting platelet function and coagulation factor expression; [40,41] FTH1 and FTL, in which the heavy and light chains of ferritin are linked to the inflammatory status, and thus may be indicators of a compromised platelet production; [42] LUM, which has been linked to hypercoagulable states in COVID19, and poses emerging roles regulating platelet function; [43] MBL2, which facilitates the interaction of platelets with the complement system in thromboinflammation; [44] and ORM1, with multiple modulatory activities, has also been suggested as a platelet inhibitor; [45] amongst others.

All these results taken together point to an altered plasma composition, which might interfere with platelet functionality. The direct interference that other molecules, alone or in combination, might exert on platelet function cannot be inferred from our analysis.

### 3.6. The Plasma Inhibitory Effects on Platelet Functionality Are Restored Depending on Treatment/Disease Progression

The observation that the plasma milieu from AML patients does exert an inhibitory effect on healthy donor platelet functionality led us to focus on treatment and disease progression. For that purpose, we plotted, as shown in Figure S3, the platelet aggregation (AUC) obtained per agonist in the presence of AML plasma, in a longitudinal manner per patient.

We had data from three patients following Induction regimens, one patient with Consolidation regimen, and four patients with Conditioning regimens prior to stem cell transplantation. For most of these patients there were samples spanning a follow-up period of about 15 days, where there were observed fluctuations, but not much variation in the plasmas inhibitory (or partially inhibitory) effects. However, we had a longer follow-up period for two patients following Induction regimens (#52 and #61). Interestingly, in these two patients, from 15 days onwards approximately, the inhibitory effects of their plasma on platelet aggregation seem to be gradually lost. Of note, one of the patients still required PLT transfusions (#61, based on PLT count).

This observation suggests that, for those patients, the plasma milieu is restored, allowing normal platelet functionality.

## 4. Discussion

In the present manuscript we describe that the plasma from AML patients exerts an inhibitory effect on healthy donor platelet functionality. The inhibitory effect is partial, and it affects most activation pathways tested, except convulxin-mediated responses. The preservation of convulxin (CVX)-induced responses suggests that the core GPVI signaling axis remains functionally intact. CVX is a potent and highly selective GPVI agonist that directly induces receptor clustering, FcRγ phosphorylation, and robust activation of the ITAM–Syk–PLCγ2 pathway [46,47]. This mechanism is largely independent of integrin cooperation or collagen structural organization. In contrast, collagen-induced platelet activation requires coordinated engagement of both GPVI and integrin α2β1 [48,49]. Moreover, collagen signaling relies more heavily on secondary amplification pathways mediated by ADP and thromboxane A_2_ [47]. Therefore, alterations affecting integrin function, receptor cooperation, or secondary feedback mechanisms could account for the impaired responses to collagen and other agonists, whereas the strong GPVI stimulus provided by CVX may overcome an increased platelet activation threshold. Remarkably, incubation with AML plasmas induced faster aggregation responses towards stimulation with Ristocetin and PMA, targeting the so-called hemostatic receptors vWF R and αIIbβ3, respectively. These alterations affecting the extent and kinetics of the aggregation capacity of donor platelets in the presence of AML plasma explain, at least in part, the variable efficacy of platelet transfusions in these patients. Of note, the functional profile of platelet responses towards the battery of agonists employed has individual particularities, and we have not included in our study other platelet functional aspects such as degranulation.

Interestingly, fluctuations were evident when studying the evolution of the effect of AML plasmas on platelet function in a longitudinal manner, per patient, during time, through a given treatment regimen. Specifically, in patients with extended follow-up, a loss of the plasma’s inhibitory effect resulting in restored platelet function was observed. This suggests that the loss of this inhibition may signify an improved prognosis for the efficacy of platelet transfusions in preventing bleeding, thereby indicating that monitoring this parameter could optimize transfusion management. To confirm these findings, future studies should utilize a larger, more diverse patient cohort with broader treatment regimen representation and sufficiently long, consistent follow-up, while integrating critical clinical endpoints such as bleeding.

Importantly, we observed distinct patterns of glycoproteins when running a selection of plasmas from AML patients on SDS-PAGE gels. These patterns align with the measured platelet aggregation profiles. While no straightforward association emerged between glycoprotein patterns and functional outcomes, cluster analysis revealed that Pattern 2 plasma was associated with the most severely impaired functional profiles.

The targeted approach made clear that plasma glycoprotein changes were associated with the aggregation inhibitory activity of the plasma. Further analysis by mass spectrometry of selected plasma glycoprotein gel bands based on the observed patterns revealed alterations in the plasma milieu. Some of these alterations referred to the location of some proteins in higher molecular bands than expected (i.e., displaced) in Patterns 2 and 3 compared to their location in the Pattern 1 sample (similar to control plasma). An explanation for this phenomenon could be the formation of multimers, the incorporation of protein complexes or protein modifications so as to run in a different height in the SDS-PAGE gels, despite the denaturing conditions. We could also identify proteins present in high molecular bands in Pattern 2 that were undetectable in the Pattern 1 sample. These proteins included FN1 and vWF, which are known to mediate in platelet functionality. While these proteins are present in all plasma samples, they may be in higher levels in those plasmas with altered glycoprotein patterns. The higher molecular weight bands characteristic of Pattern 2 likely correspond to vWF multimers, based on the mass spectrometry results, which may be responsible for the altered kinetics of PMA- and Ristocetin-mediated responses, due to direct interaction with the vWF receptor, subsequently triggering the activation of the αIIbβ3 integrin through inside-out signaling [50].

Current proteomic data from AML patients during intensive therapy show significant alterations in circulating proteins involved in the acute-phase response and coagulation, suggesting a proteomic imbalance that potentially drives the platelet inhibition reported in our study [16]. Beyond causing thrombocytopenia, chemotherapy has been shown to impair platelet functionality by reducing activation markers and integrin αIIbβ3 activation, regardless of the malignancy type or treatment regimen [51]. We recognize that definitively separating the effects of AML from chemotherapy exposure is challenging in our current patient group. Since platelet reactivity in treated patients is influenced by a complex interplay of disease and environment, we should acknowledge the absence of systematic pre-treatment sampling as a limitation. In summary, our observations suggest that the patient plasma milieu in AML contributes to the functional suppression of donor platelets, necessitating further research to decouple the specific effects of the disease characteristics and the treatment itself.

We observed increased levels (although with variation) of vWF in plasma samples of AML patients, as quantified by ELISA. The increased levels of vWF and FN1 is a common finding in leukemic patients [52,53,54]. A potential explanation for this phenomenon is the prolongation of the elimination half-life of vWF due to the loss of macrophages after cytotoxic Chemotherapy. Alterations in the glycosylation of the vWF molecule might additionally affect the elimination half-life of vWF and/or the affinity to its platelet receptor, and this might be extrapolated to other interactions. How deregulated levels and alterations in the plasma milieu affect platelet functionality cannot be ascertained from our data, and there are many factors beyond glycosylation that should be further analyzed in future studies. However, our results show that dilution of plasmas from AML patients that inhibited Ristocetin-mediated responses with plasma from AB RhD+ healthy donors, seemed sufficient to restore the inhibitory effect to that particular response.

## 5. Conclusions

Our set of data corroborates that the plasma milieu is altered in AML patients. This observation is largely acknowledged per se, given the inflammatory status that characterizes the disease and its progression through treatment, the consequent acute-phase response and the alterations in the hematopoietic production. Importantly, our study, however, pinpoints that this inhibitory variability might be caused by changes in plasma glycoproteins, amongst which important platelet adhesion and aggregation mediating molecules like vWF and fibrinogen. Considering the need to improve the efficacy of platelet transfusions in hemato-oncological patients, our results suggest that a test of the compatibility and functional interference of plasma from AML patients with healthy donor platelets might be useful to identify additional bleeding risk. Further investigation is required to identify the most important factors and possibly identify and test additional interventions to limit remaining bleeding in patients that up to now only receive prophylactic platelet transfusions. Such other adjuvant therapies might be transfusion of non-inhibitory plasma or plasmapheresis. Such strategies shall be further studied and validated in future studies.

## Data Availability

Data is available in the Supplementary Materials.

## Supplementary Materials

The following supporting information can be downloaded at: https://www.mdpi.com/article/10.3390/biom16060761/s1, Figure S1: Representative aggregation graphs obtained with AML plasma samples and control plasma. Figure S2: Photographs of gels used for mass spectrometry analysis; Figure S3: Healthy donor platelet aggregation follow-up per patient; Table S1: Mass spectrometry data; Table S2: Data contained in the manuscript per figure and patient clinical data. Gels original images.

## Abbreviations

The following abbreviations are used in this manuscript:

PLT	Platelets
AML	Acute myeloid leukemia
CCI	Corrected count increment
CRP	Collagen-related peptide
MDS	Myelodysplastic syndrome
FCA	Flow cytometry-based platelet aggregation assay (test)
PREPAReS	Pathogen Reduction Evaluation and Predictive Analytical Rating Score trial
AUC	Area under the curve
TMA	Time of maximum aggregation
vWF	Von Willebrand factor
MS	Mass spectrometry
SD	Standard deviation
