# Supplementary material for: The Plasma Glycoprotein Milieu in the Hemato-Oncological Patient Inhibits Platelet Function
Source: Biomolecules. 2026 May 22;16(6):761. doi: 10.3390/biom16060761 (PMC13296933; doi:10.3390/biom16060761)
Supplement: Supplementary file 1 [file biomolecules-16-00761-s001.zip › Carbajo Arguelles et al may 26 Supp Figures and Legends.pdf]

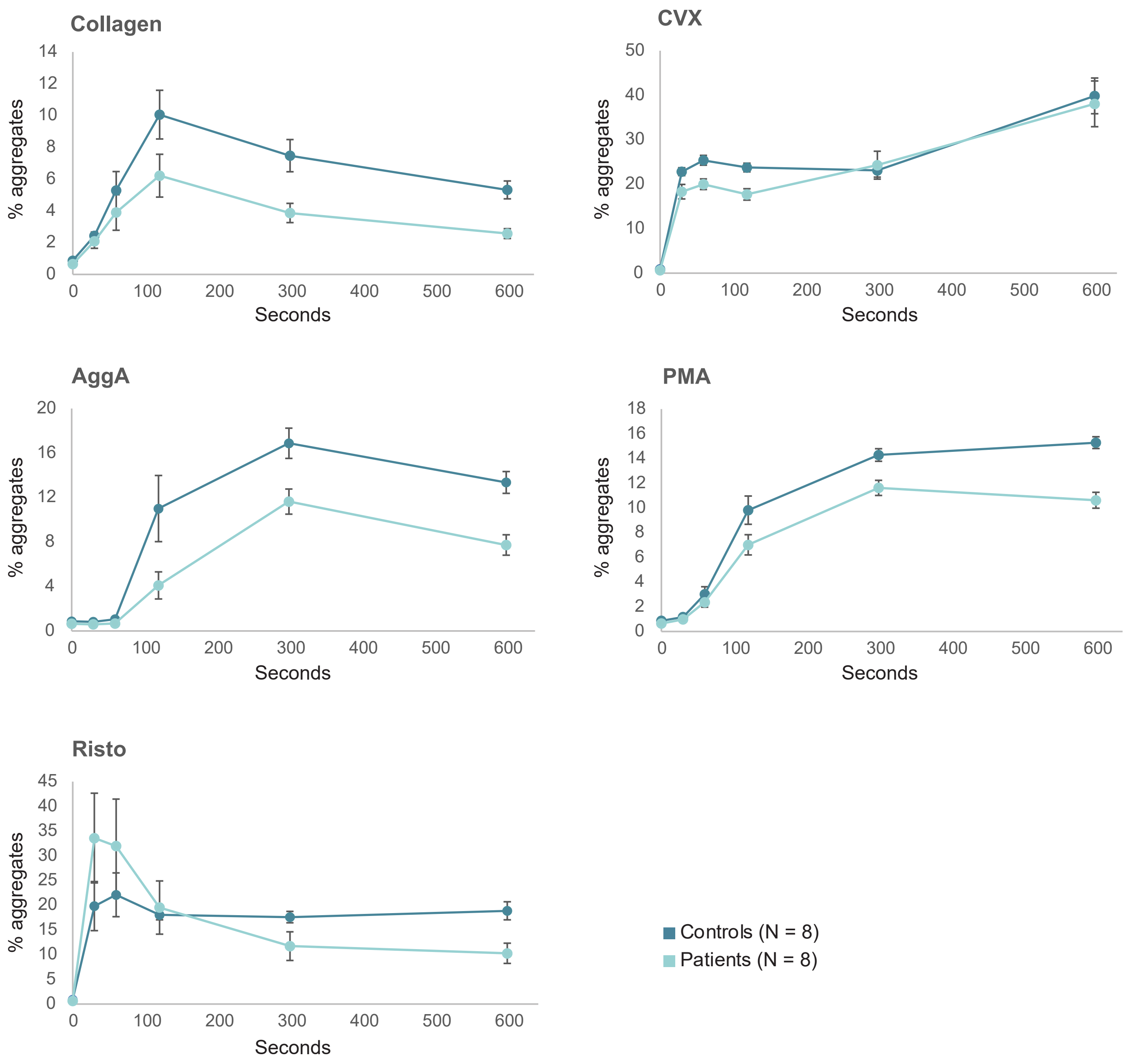

Collagen
CVX
AggA
PMA
Risto
% aggregates
Seconds
Controls (N = 8)
Patients (N = 8)

Figure S1

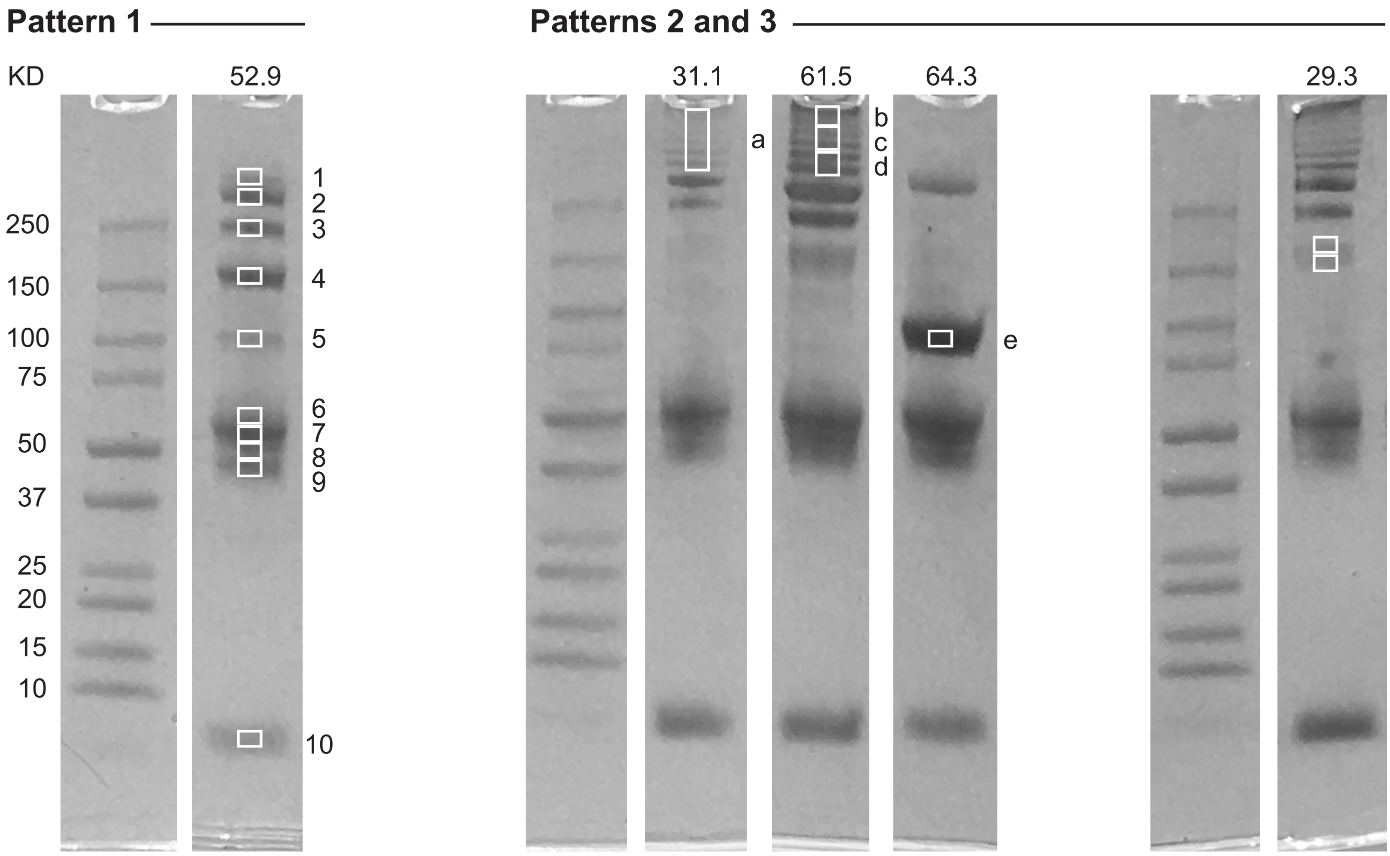

Pattern 1
Patterns 2 and 3
KD
52.9
31.1
61.5
64.3
29.3
250
150
100
75
50
37
25
20
15
10
1
2
3
4
5
6
7
8
9
10
a
b
c
d
e
f
g

Figure S2

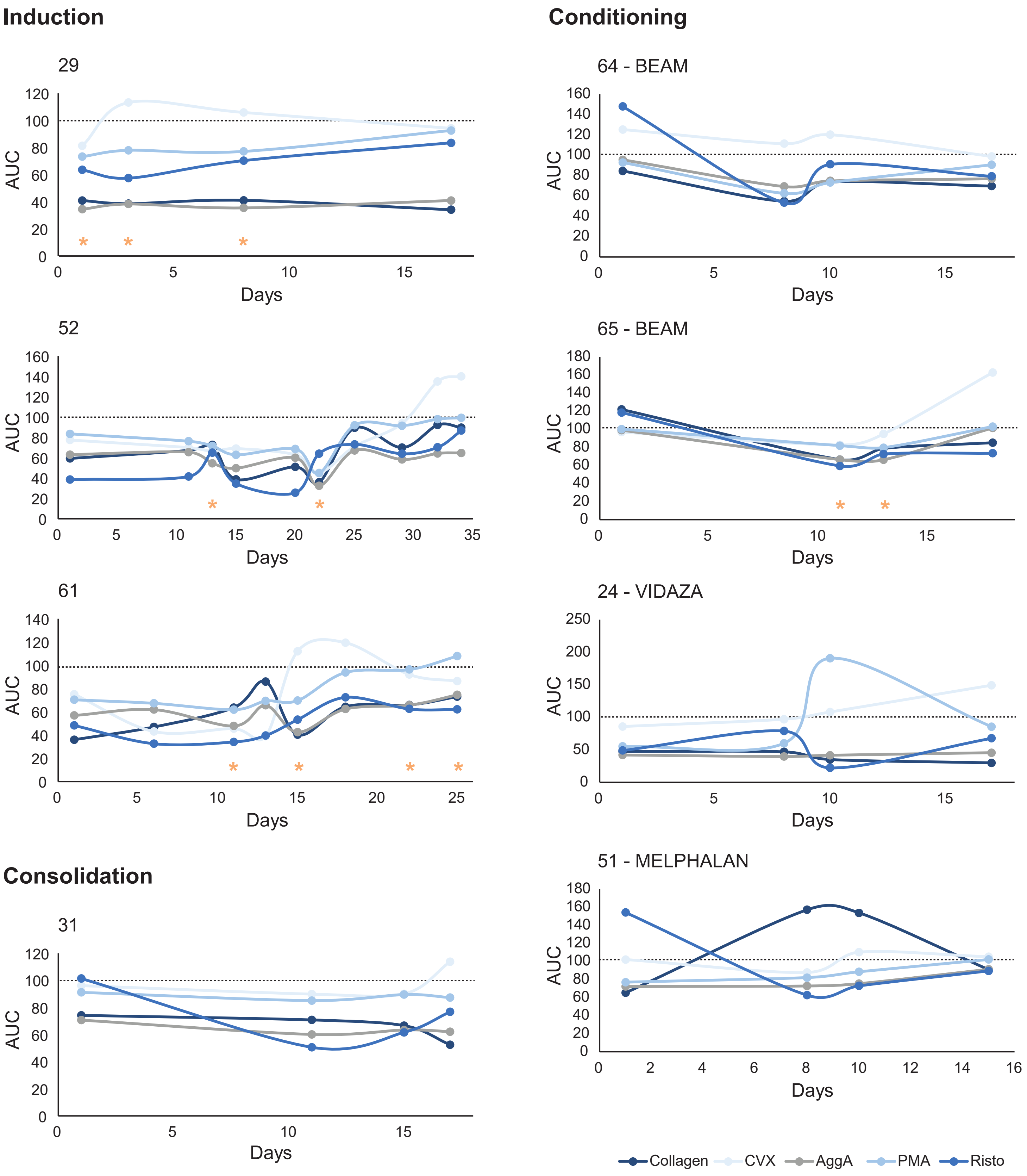
Induction
29
52
61
Consolidation
31
Conditioning
64 - BEAM
65 - BEAM
24 - VIDAZA
51 - MELPHALAN
AUC
Days
Collagen
CVX
AggA
PMA
Risto

Figure S3

Supplementary Figure Legends

Figure S1: Representative platelet aggregation graphs

The raw platelet aggregation graphs towards all agonists tested from representative reactions performed with healthy donor platelets in the presence of either control plasma (N = 8) or AML plasma samples (N = 8) are depicted. Average and standard error of the mean (SEM) are represented. CVX, convulxin; AggA, Aggretin A, PMA, Phorbol 12-myristate 13-acetate; Risto, ristocetin.

Figure S2: Photographs of gels used for mass spectrometry analysis

Selected samples from Patterns 1-3 were run on SDS-PAGE gels and stained for glycoproteins. White squares indicate the cut gel band portions used for mass spectrometry analysis. KD, kiloDalton.

Figure S3: Healthy donor platelet aggregation follow-up per patient

Line graphs depicting the healthy donor platelet aggregation responses (Area under the curve -AUC-) through time, per patient. The treatment regimens are indicated. Each colored line represents the aggregation response towards a different agonist. Data has been normalized, setting responses in the presence of AB RhD^+ control plasma to 100. Asterisks indicate when a platelet transfusion was given to a patient, although the data represented is obtained with AML plasmas pre-transfusion. CVX, convulxin; AggA, Aggretin A, PMA, Phorbol 12-myristate 13-acetate; Risto, ristocetin.
