# Supplementary figures and images for: The Plasma Glycoprotein Milieu in the Hemato-Oncological Patient Inhibits Platelet Function
Source: Biomolecules. 2026 May 22;16(6):761. doi: 10.3390/biom16060761 (PMC13296933; doi:10.3390/biom16060761)

GEL 3

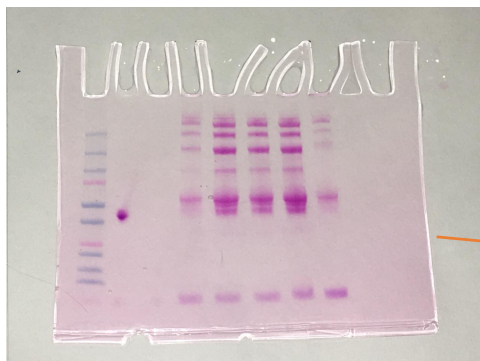

A

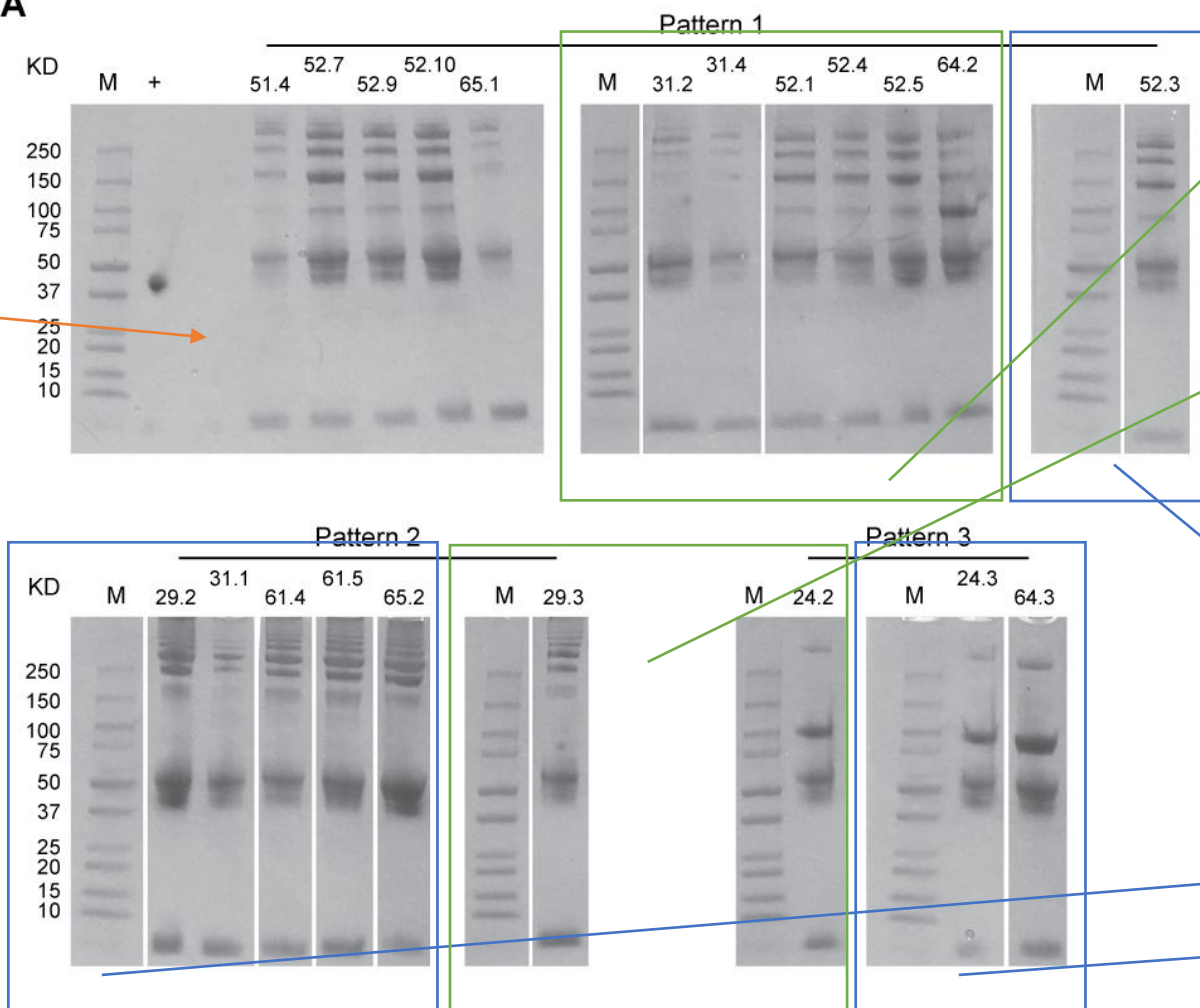

GEL 1

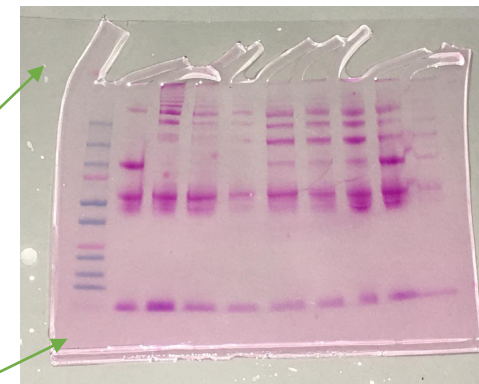

GEL 2

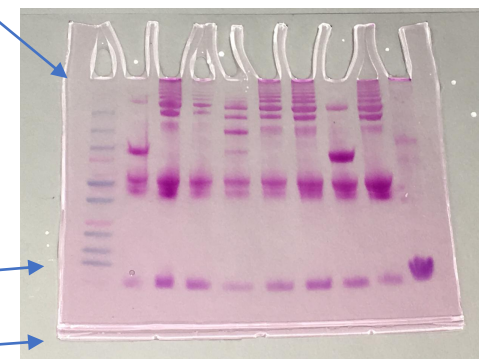

Pattern 1

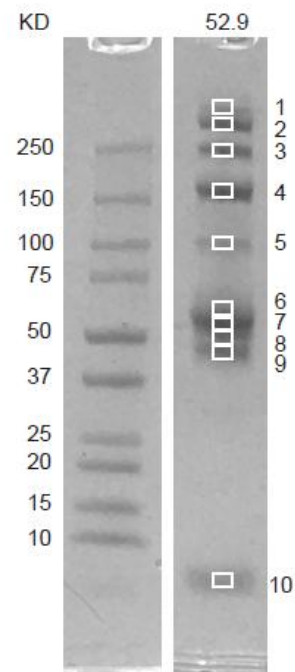

Figure S1

Patterns 2 and 3

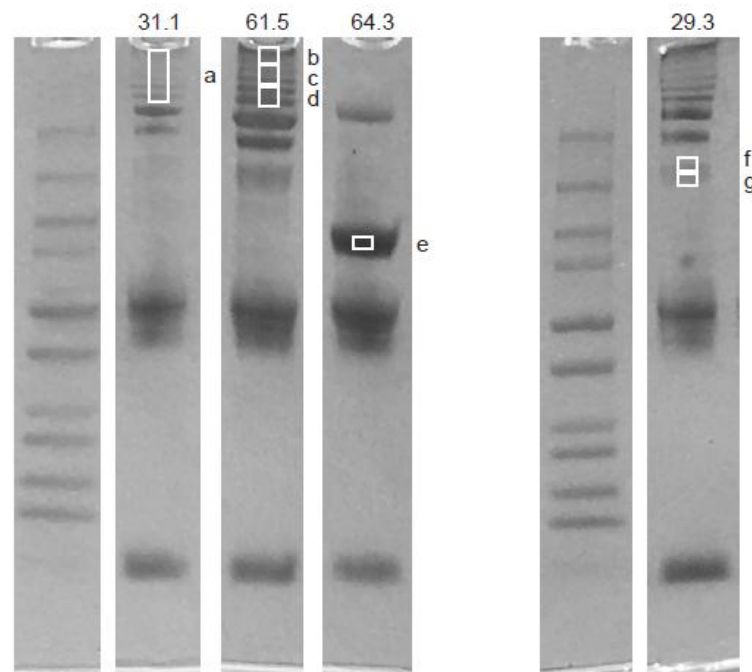

GEL 2

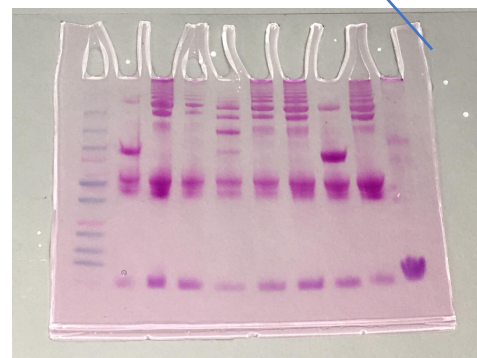

GEL 1

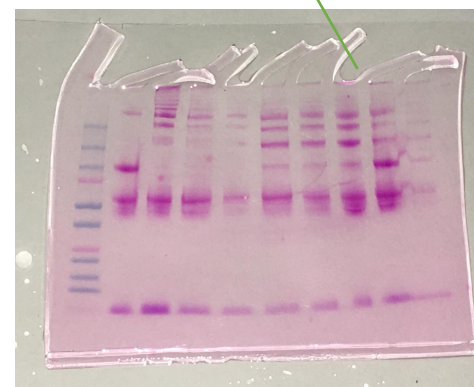

GEL 3

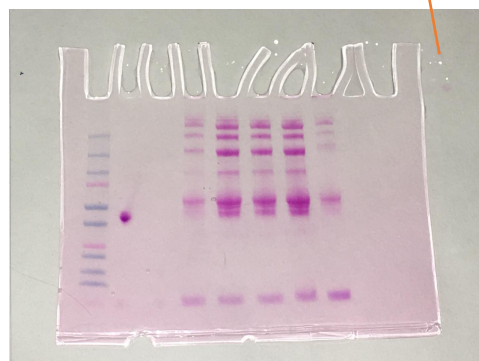

# GEL 1

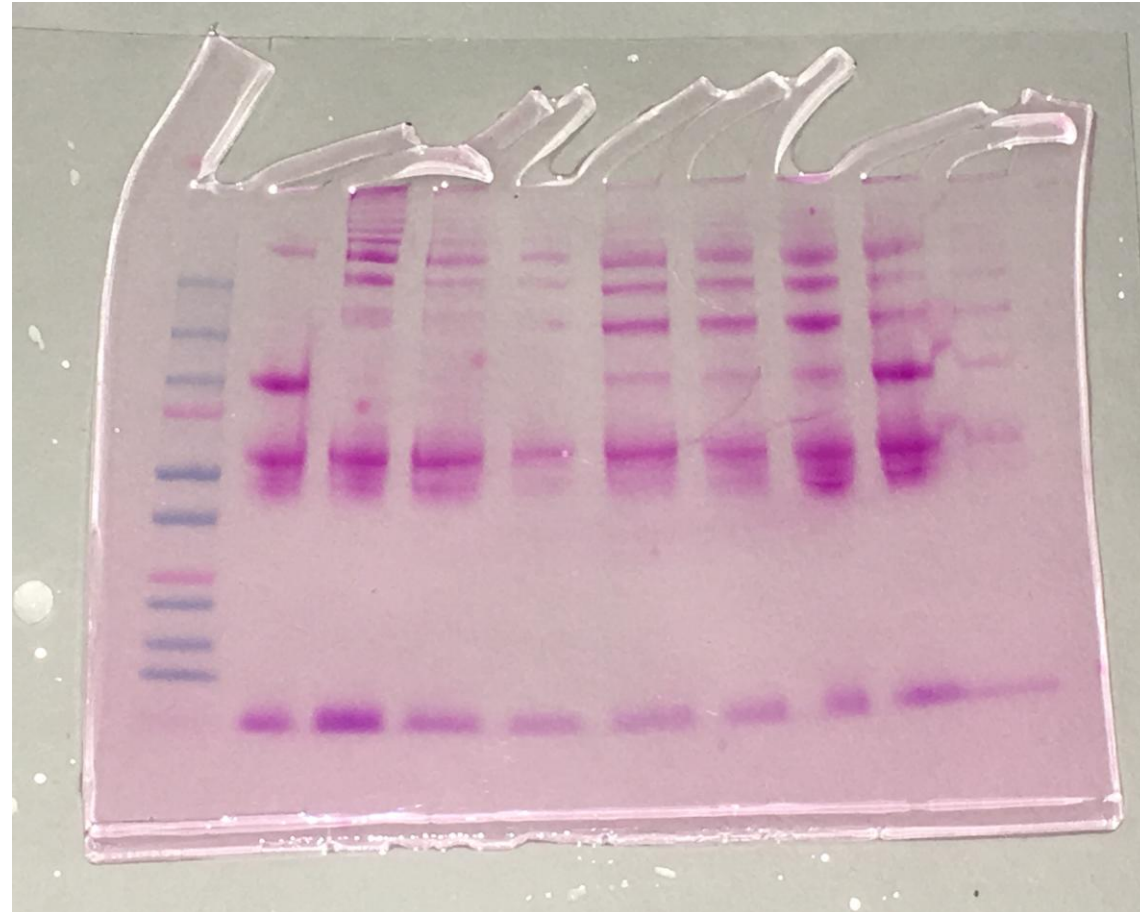

# GEL 2

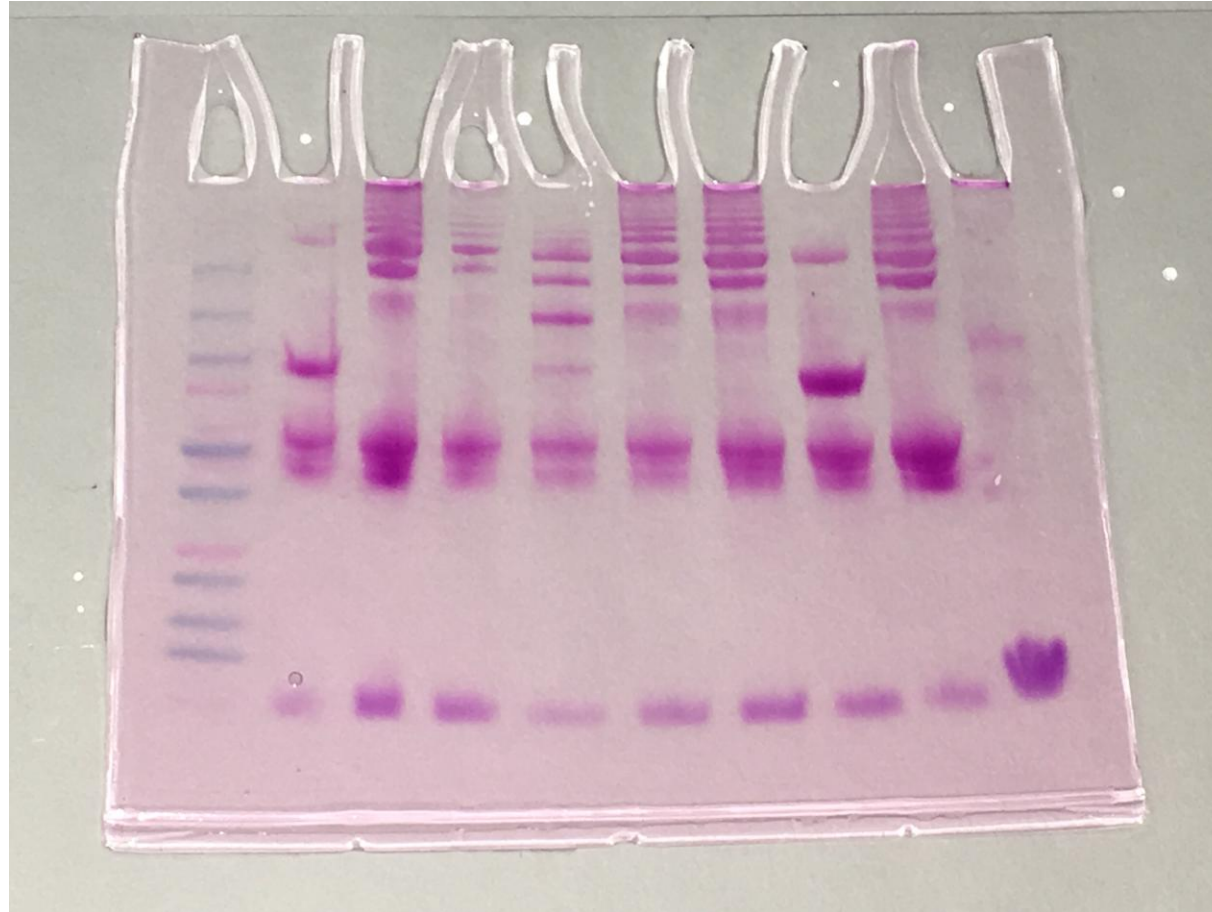

# GEL 3

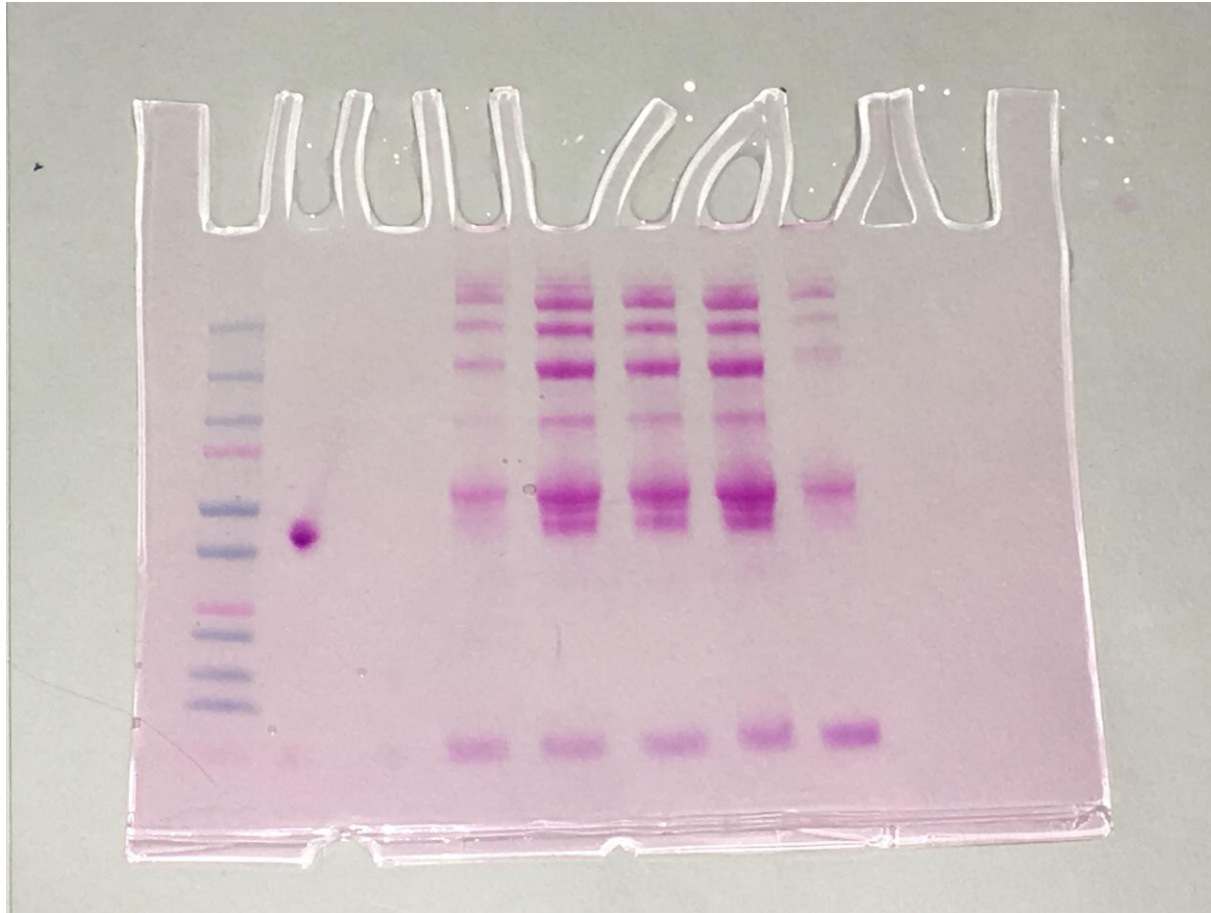

Supplement: Supplementary file 1 [file biomolecules-16-00761-s001.zip › Explanation GELS original images.pdf]
